# Comparison of Electrocardiogram between Dilated Cardiomyopathy and Ischemic Cardiomyopathy Based on Empirical Mode Decomposition and Variational Mode Decomposition

**DOI:** 10.3390/bioengineering11101012

**Published:** 2024-10-11

**Authors:** Yuduan Han, Chonglong Ding, Shuo Yang, Yingfeng Ge, Jianan Yin, Yunyue Zhao, Jinxin Zhang

**Affiliations:** 1 Department of Medical Statistics, School of Public Health, Sun Yat-sen University, Guangzhou 510080, China; hanyd5@mail2.sysu.edu.cn (Y.H.); dingchlong@mail2.sysu.edu.cn (C.D.); yangsh223@mail2.sysu.edu.cn (S.Y.); geyf8@mail2.sysu.edu.cn (Y.G.); yinjn@mail2.sysu.edu.cn (J.Y.); 2 Union Hospital, Tongji Medical College, Huazhong University of Science and Technology, Wuhan 430022, China; 3 Department of Cardiology, The Third Affiliated Hospital of Sun Yat-sen University, Guangzhou 510630, China

**Keywords:** dilated cardiomyopathy, ischemic cardiomyopathy, electrocardiogram, variational mode decomposition, bispectral analysis

## Abstract

The clinical manifestations of ischemic cardiomyopathy (ICM) bear resemblance to dilated cardiomyopathy (DCM), yet their treatments and prognoses are quite different. Early differentiation between these conditions yields positive outcomes, but the gold standard (coronary angiography) is invasive. The potential use of ECG signals based on variational mode decomposition (VMD) as an alternative remains underexplored. An ECG dataset containing 87 subjects (44 DCM, 43 ICM) is pre-processed for denoising and heartbeat division. Firstly, the ECG signal is processed by empirical mode decomposition (EMD) and VMD. And then, five modes are determined by correlation analysis. Secondly, bispectral analysis is conducted on these modes, extracting corresponding bispectral and nonlinear features. Finally, the features are processed using five machine learning classification models, and a comparative assessment of their classification efficacy is facilitated. The results show that the technique proposed provides a better categorization for DCM and ICM using ECG signals compared to previous approaches, with a highest classification accuracy of 98.30%. Moreover, VMD consistently outperforms EMD under diverse conditions such as different modes, leads, and classifiers. The superiority of VMD on ECG analysis is verified.

## 1. Introduction

Dilated cardiomyopathy (DCM) and ischemic cardiomyopathy (ICM) present with highly similar clinical manifestations, standing as the predominant etiologies of heart failure [1]. However, their treatments and prognoses diverge significantly. The treatment of DCM focuses on reducing cardiac load and promoting cardiac contraction [2]. Conversely, the treatment for ICM usually focuses on restoring the patency of the coronary arteries to improve the blood supply to the heart muscle [3]. Hence, an early and precise differential diagnosis between DCM and ICM exerts a positive influence on the subsequence [4].

The gold standard for clinically diagnosing both diseases is coronary angiography [5]. Nonetheless, challenges are posed by the invasive nature, high cost, stringent personnel and equipment requirements, and associated contraindications. In contrast, Electrocardiogram (ECG) is an electrophysiological method for recording the cardiac activity [6]. It has gained wide application in clinical settings owing to its non-invasiveness, affordability and convenient operation [7]. However, the recording of procedures by different technicians, with the interpretative skills of physicians, frequently yields divergent results, due to variances in educational backgrounds, training, and clinical experiences [8].

With signal processing technology advancing, artificial intelligence is increasingly utilized for ECG signal processing, extracting information features from various perspectives [9]. Researchers have recently analyzed ECG signals using mode decomposition and machine learning algorithms, gaining a deeper understanding of associated diseases and underlying mechanisms of change [10,11,12].

Empirical Mode Decomposition (EMD) was introduced by N. E. Huang in 1998, and constitutes a fundamental component of the Hilbert transform. This method is adept at handling nonlinear, non-stationary signals in both the time and frequency domains [13]. The signal can be decomposed into several stable components based on its inherent characteristics. In maternal–fetal ECG signal analysis, pure data are extracted from the baseline based on EMD with excellent performance and improved signal-to-noise ratios [14]. Several researchers have applied EMD to single-lead ECG signals in the classification of myocardial ischemia and non-ischemia, extracting distinctive features and achieving a classification sensitivity of 88% [15]. Furthermore, EMD has been widely used in the identification of atrial fibrillation [16] and estimation of fetal heart rate [17], and automatic classification of ECG signals.

Variational mode decomposition (VMD), an innovative technique for signal decomposition, was recently introduced by Dragomiretskiy and Zosso [18]. Compared with EMD, the modes derived from VMD exhibit reduced susceptibility to noise interference and are underpinned by appropriate mathematical models. Due to its excellent performance in the fields of biological and speech signal processing, VMD has been widely used in these fields, showing superior efficacy compared to EMD [19]. In the realm of ECG processing, many studies have utilized VMD’s ability to capture local variations of clinical components by exploiting the morphological similarities between the mode and the QRS complex [20]. In studies related to sleep apnea, ECG signals have been identified through modal decomposition and machine learning algorithms [19]. Similarly, a study proposed a VMD classification technology based on the automatic detection of single-lead ECG signals to identify ventricular premature beats and sinus rhythm, and reported an average classification accuracy of 98.72% [21]. Additionally, a study focused on atrial fibrillation recognition employed VMD mode to capture features, and the highest accuracy is 98.27% [22].

In this study, the ECG signals are analyzed to distinguish between DCM and ICM, ultimately aiming to offer valuable insights for clinical practice. On one hand, the differentiation between DCM and ICM based on ECG signals seeks to provide a definitive diagnosis, improve medication planning, and improve the overall prognosis for individuals with undetermined cardiomyopathy. On the other hand, EMD and VMD have not been extensively explored in ECG. This study aims to address this research gap by comparing the two methods in differential diagnosis of DCM and ICM in ECG.

## 2. Methodology

**Figure 1:**
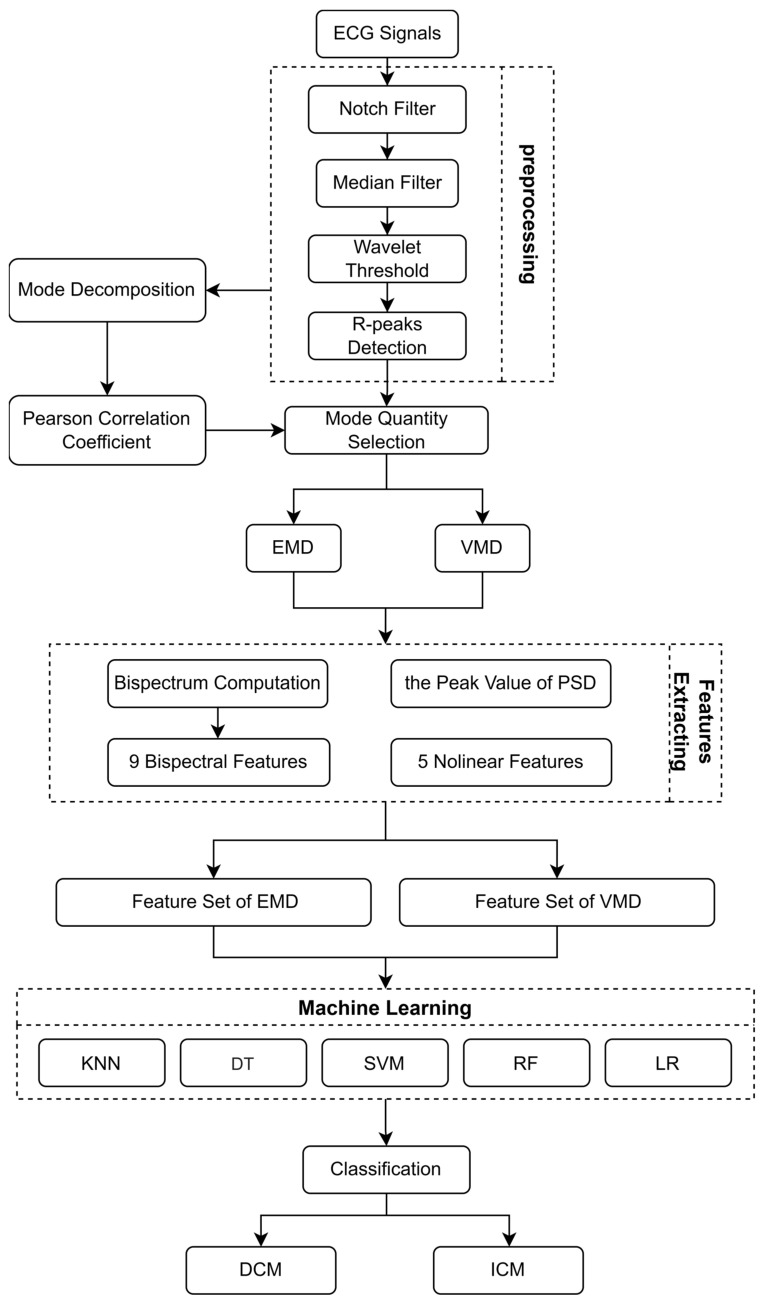
Overview diagram of ECG signals classification.

The diagram of the proposed method is shown in Figure 1.

### 2.1. Dataset

The data utilized in this study were sourced from the Department of Cardiovascular Medicine at the Third Affiliated Hospital of Sun Yat-sen University. In total, 87 patients who visited the hospital between 1 January 2016, and 30 September 2023, met the inclusion and exclusion criteria and were included in the study. The collected information was reviewed by an experienced cardiovascular physician to ensure data quality. This study was approved by the Ethics Committee of the School of Public Health of Sun Yat-sen University. Because of the retrospective design with anonymized data, an exemption status for individual informed consent was provided.

Participants were divided into two groups: ICM, 43 patients; DCM, 44 patients. The inclusion and exclusion criteria are as follows [3]: ① Left Ventricular Ejection Fraction was less than 45%; ② the inner diameter of the left ventricle at the end of diastole was greater than 55 mm in males and 50 mm in females; ③ patients with DCM were identified by coronary angiography as having left main duct lumen stenosis < 50% and epicardial coronary lumen < 75%; ④ patients with ICM were identified by coronary angiography as having left trunk lumen stenosis ≥ 50% or epicardial coronary lumen ≥ 75%. Exclusion criteria: ① pleural effusion; ② lung tumor; ③ history of myocardial infarction; ④ permanent pacemaker implantation.

All patients underwent ECG and echocardiography within 24 h before coronary angiography. The ECG-3312 machine of Guangzhou SanRui Electronic Technology Co., Ltd.(Guangzhou, China) was used with ECGNET-V3.10 ECG data management software (ECGNET-V3.10) to collect the signals of 12 leads (I, II, III, aVR, aVL, aVF, V1, V2, V3, V4, V5, V6), and the sampling frequency was 1000 Hz. In this study, ECG records of each subject were selected for further processing and analyzing. Table 1 shows baseline characteristics of patients in DCM and ICM.

### 2.2. Preprocessing

ECG is susceptible to different types of noise, such as power-line interference [23], baseline wander [24], and muscle artifacts [25]. In this study, we apply the 50 Hz notch filter, the median filter, and wavelet threshold denoising to mitigate these interferences.

**Figure 2:**
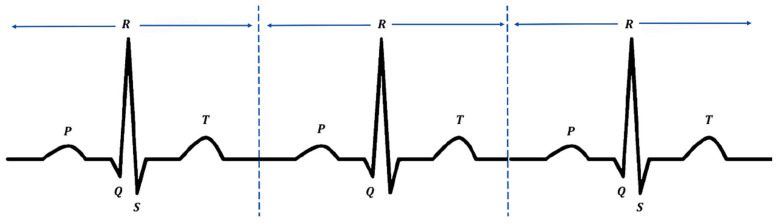
A diagram of the heartbeats divided by R-peaks.

To achieve data standardization, ECG signals are fragmented into multiple tiny segments, where each represents one heartbeat. The Pan–Tompkins algorithm is used to identify the R-peaks. With its straightforward computation and simple implementation, this approach is reliable for R-peak identification [26]. A series of 300 samples before a QRS peak, 300 samples after the peak, and the QRS peak itself are consolidated into a 601-sample segment for subsequent analysis after detecting the QRS peak [27]. We exclude the initial and final beats from the entire dataset to ensure a consistent count of 601 sample points. Figure 2 shows a diagram of the heartbeat pattern divided by R-peak. A total of 6533 heart beats (3639 DCM and 2894 ICM) were included in the subsequent analysis.

### 2.3. Empirical Mode Decomposition

EMD is a method to decompose any time series into a set of intrinsic mode functions (IMFs), whether nonstationary or nonlinear.

**Figure 3:**
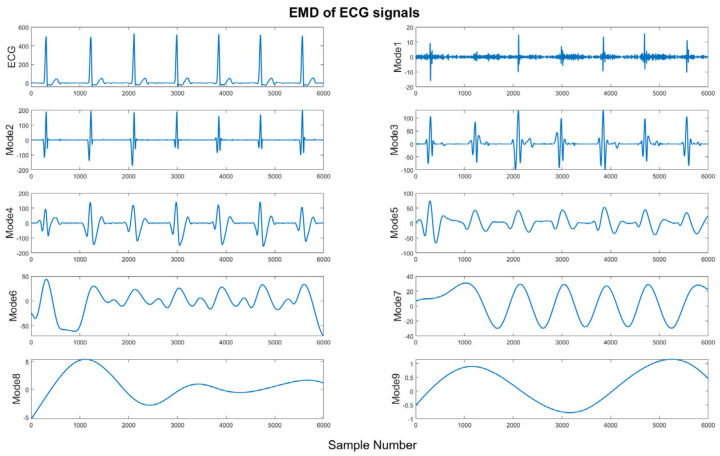
Illustration of nine modes of EMD.

The original ECG signal $\left\{f\left(t\right):t=1,\;2,\;3,\;\ldots ,\;N\right\}$ can be expressed as follows:

(1) $$f\left(t\right)=\sum_{i=1}^{M}{imf}_{i}\left(t\right)+r_{M+1}(t)$$

where ${imf}_{i}$ is the *i*th eigenmode function, *M* is the number of all eigenmode functions, and ${\;r}_{M+1}\left(t\right)$ is the final residual, representing the average trend in the original signal. Figure 3 shows a sample of the nine modes based on the EMD of ECG signals.

### 2.4. Variational Mode Decomposition

**Figure 4:**
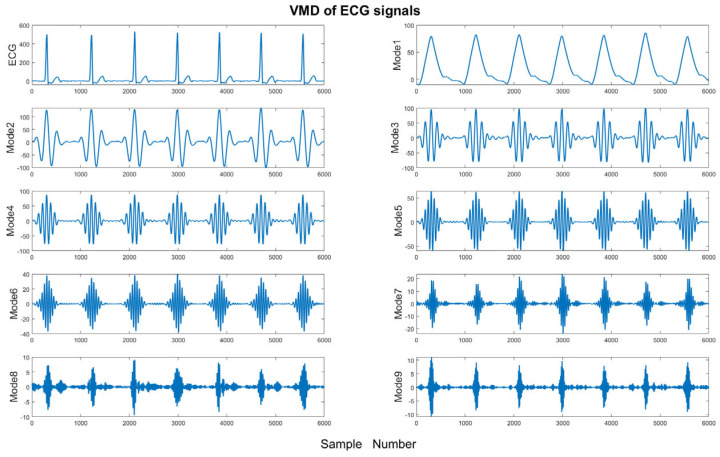
Illustration of nine modes of VMD.

The VMD constrained problem is mathematically defined as:

(2) $$\underset{\left\{u_{k}\},\{\omega_{k}\right\}}{min}\left\{\sum_{k=1}^{K}{\left\|{𝜕}_{t}\left[\left(\delta (t)+\frac{i}{\pi t}\right)*u_{k}(t)\right]e^{-i\omega_{k}t}\right\|}_{2}^{2}\right\},\;s.t.\sum_{k=1}^{K}u_{k}(t)=f$$

where $\left\{u_{k}\right\}≔\{u_{1},\;u_{2},\;\ldots ,\;u_{K}\}$ and $\;\left\{\omega_{k}\right\}≔\left\{\omega_{1},\;\omega_{2},\;\ldots ,\;\omega_{K}\right\}\;$ reflect shorthand notations for the *k*th mode of the ECG signal and their center frequencies. Figure 4 shows a sample of the nine modes based on the VMD of ECG signals.

### 2.5. Mode Quantity Selection

The signal obtained by the mode decomposition method is a series of decomposed signals which cannot be directly used for classification because of the high feature dimensions. To solve this problem, the Pearson correlation coefficient is used to measure the linear correlation between the first ten modes $\left\{x_{k}(t)\right\}≔\left\{x_{1}\left(t\right),\;x_{2}\left(t\right),\;\ldots ,\;x_{k}\left(t\right)\right|\;k=1,\;2,\;\ldots ,\;10,\;t=1,\;2,\;\ldots ,\;N\}$ and the original ECG signal. In the field of natural science, the Pearson correlation coefficient is widely used to measure the degree of correlation between two variables [28]. The Pearson correlation coefficient between the *k*-mode of lead *j* and its original ECG signal $\left\{f_{i}\left(t\right):t=1,\;2,\;\ldots ,\;N\right\}$ in case *i* is $r_{ijk}$ as follows:

(3) $$r_{ijk}=\frac{\sum_{t=1}^{N}\left(x_{ijk}(t)-{\bar{x}}_{ijk}(t))(f_{ij}(t)-{\bar{f}}_{ij}(t)\right)}{\sqrt{\sum_{t=1}^{N}(x_{ijk}(t)-{\bar{x}}_{ijk}(t){)}^{2}}\sqrt{\sum_{t=1}^{N}(f_{ij}(t)-{\bar{f}}_{ij}(t){)}^{2}}}$$

Here $x_{ijk}(t)$ is the *k*-mode in lead *j* in case *i*, and *t* denotes the sampling points of the ECG signal. In this study, the Pearson correlation coefficients of the ECG signal and the decomposed modes in different leads were calculated, and then the mean value ${\bar{r}}_{\cdot jk}$ was used as the energy correlation coefficient, and the number of modes was selected as the reference for the energy correlation coefficients between the *j*-th lead and the first ten modes (*k* = 1, …, 10).

Modes with higher correlation coefficients are more correlated with the original signal, indicating concentrated signal energy in these modes. In this study, correlation coefficients between each lead and the first ten modes were used to determine the number of modes, selecting those with more concentrated energy for further analysis. Subsequently, correspondence analysis [29] was used to generate the factor loading plot [30,31], visualizing the correspondence between leads and modes.

### 2.6. Bispectrum Computation and Feature Extraction

High order spectrum analysis is widely used to extract subtle changes in biological signals. Compared with a low-order spectrum, a high-order spectrum can provide more accurate signal estimation and analysis [32], as the higher-order moments and cumulants of the modes are observed [33]. In this study, we used the third-order statistic (bispectrum) to analyze the modes, calculate the high-order cumulant of ECG signal, and analyze the graph of the high-order cumulant.

The *x*(*n*) third-order cumulant is defined as follows:

(4) $$R_{3x}(\tau_{1},\tau_{2})=E\left[x(n)x(n+\tau_{1})x(n+\tau_{2})\right]$$

where $\tau_{1}$ and $\tau_{2}$ represent time shift. E[·] represents mathematical expectations.

The bispectrum of *x*(*n*) is:

(5) $$B_{x}(f_{1},\;f_{2})=\sum_{\tau_{1}=-\infty }^{+\infty }\sum_{\tau_{2}=-\infty }^{+\infty }R_{3x}(\tau_{1},\tau_{2})\cdot e^{-j(f_{1}\tau_{1}+f_{2}\tau_{2})},\;(\left|f_{1}\right|,\left|f_{2}\right|\leq \pi )$$

where $f_{1}$ and $f_{2}$ are two independent frequencies. In this paper, the third-order periodic-method discrete Fourier transform is used to directly estimate the bispectrum based on the decomposed modes.

To avoid losing information, this study extracted information directly from the bispectral matrix [34]. In this study, a total of 15 features, including bispectral flatness, bispectral brightness, and bispectral roll-off, etc. [35,36,37,38], were extracted and incorporated into the model, as detailed in Table 2.

### 2.7. Classification

In this study, five common machine learning classification algorithms were employed to determine the optimal classifier, including logistic regression(LR), support vector machine(SVM) [39], decision tree(DT) [40], random forest(RF) [41], and *K*-Nearest Neighbor (KNN) [42]. A 10-fold cross validation approach was implemented to mitigate classifier overlap during training and testing. This study evaluated model discriminant ability using accuracy (*ACC*), sensitivity (*SEN*), specificity (*SPE*), positive predictive value (*PPV*), negative predictive value (*NPV*), and area under the curves (*AUC*).

(6) $$ACC=\frac{TP+TN}{TP+FP+TN+FN}$$

(7) $$SEN=\frac{TP}{TP+FN}$$

(8) $$SPE=\frac{TN}{FP+TN}$$

(9) $$PPV=\frac{TP}{TP+FP}$$

(10) $$NPV=\frac{TN}{TN+FN}$$

where *TP* is the classifier successfully classifies it into the DCM group when the label of the input ECG signal is DCM. *TN* is the classifier successfully classifies it into the ICM group when the label of the input ECG signal is ICM. *FP* is the classifier wrongly classifies it into the DCM group when the label of the input ECG signal is ICM. *FN* is the classifier wrongly classifies it into the ICM group when the label of the input ECG signal is DCM.

### 2.8. Model Interpretation and Feature Importance

The SHAP value was introduced in this study. SHAP is a unified framework proposed by Lundberg and Lee [43] to interpret machine learning predictions, and it is a new approach to calculate the importance of ranking features from the model. We leveraged SHAP to provide a variable importance ranking for our predictive model.

## 3. Results

**Figure 5:**
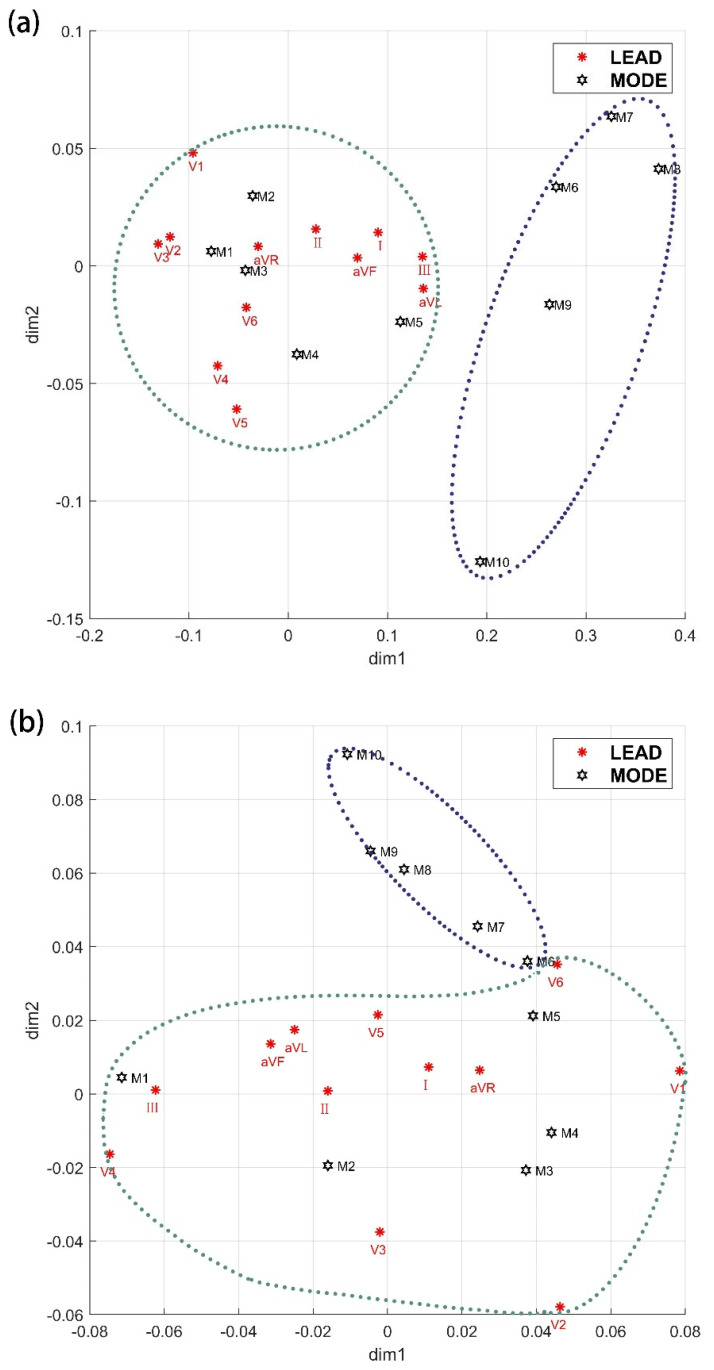
Factor loading by corresponding analysis. (**a**) Factor loading by corresponding analysis for EMD; (**b**) Factor loading by corresponding analysis for VMD.

In this study, Pearson correlation coefficients were calculated between the modes and the original signal, as well as between adjacent modes. Table 3 shows the Pearson correlation coefficients between the first ten modes and the original EMD and VMD signals. Figure 5 shows the corresponding analysis-factor load diagram for EMD and VMD.

Under each lead, the correlation coefficients between the more frontward modes and the original signal are higher, while those between the posterior modes and the original signal are lower. Therefore, the initial 5 modes were selected to retain modes with more energy information from the original signal, ensuring result reliability [21]. Thus, keeping the initial 5 modes in this study prevents energy information overlap.

Table S1 shows that the cumulative contribution rate of both dimensions in the correspondence analysis exceeds 90%, supporting the application of two-dimensional correspondence analysis. According to the biplots from corresponding analysis for EMD and VMD in Figure 5, the initial five modes exhibit close adjacency to the 12 leads, whereas the last five modes are independent of the 12 leads. From a visualization standpoint, the scientific validity of extracting the initial five modes in this study is corroborated.

**Figure 6:**
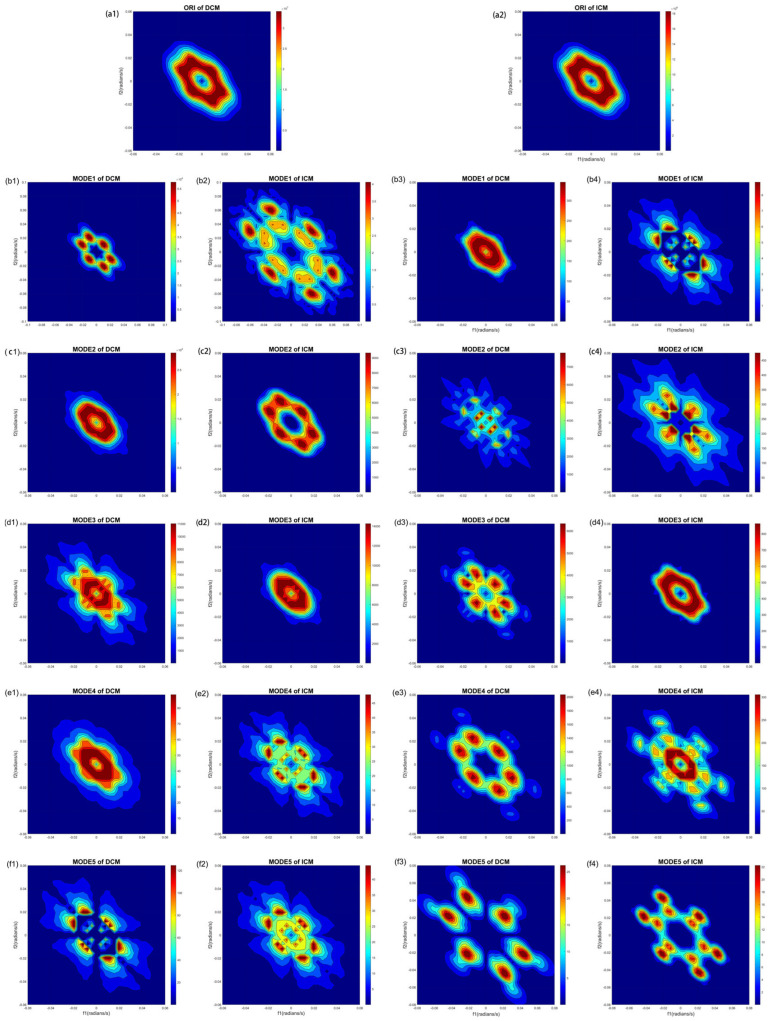
The contour bispectrum of DCM and ICM by EMD and VMD. (**a1**) is the original ECG of DCM; (**a2**) is the original ECG of ICM; (**b1**–**f1**) are the ECG mode of DCM by EMD; (**b2**–**f2**) are the ECG mode of ICM by EMD; (**b3**–**f3**) are the ECG mode of DCM by VMD; (**b4**–**f4**) are the ECG mode of ICM by VMD.

Figure 6 shows the average bispectrum of DCM and ICM within the frameworks of EMD and VMD, respectively. As illustrated in Figure 6(a1,a2), the differences in bispectrum between DCM and ICM are manifested in the magnitude of amplitude values. The bispectrum amplitude for DCM is on the order of 10^7^, surpassing the ICM amplitude, which is on the order of 10^6^. Under both EMD and VMD frameworks, DCM consistently shows higher bispectrum amplitude magnitudes than ICM, reflecting differences across various modes.

Table 4 shows the comparison of the highest classification effects of the five classifiers in each lead under the EMD and VMD frameworks. The classification effect of each classifier by VMD is better than EMD. Under EMD, RF showed the best classification effect in each lead, followed by KNN, SVM, LR, and DT classification effects being the worst. Under VMD, RF, and KNN the best classification effect was had, followed by SVM, and DT being slightly better than LR. Both RF and KNN showed higher classification performance under the two decomposition frameworks, while LR showed poor classification effect. And the highest classification accuracy, 98.3%, was achieved using the V3 lead under the VMD framework. The classification performance of five classifiers under the EMD and VMD frameworks were shown in Tables S2 and S3. And the grouping box diagrams of the average accuracy of each mode of 12 leads under EMD and VMD were shown in Figures S2 and S3.

In this study, the multivariate analysis of variance (MANOVA) is performed on the five classifiers to test the difference in classification index mean vectors of five classifiers under EMD and VMD frameworks. Given that $\mu_{i}$ (*i* = 1, 2, …, 5) is a 12 × 1 mean vector composed of the same classification index in 12 leads. The null hypothesis is $H_{0}:\mu_{1}=\cdots =\mu_{5}$. Then, Hotelling *T*^2^ test is used to identify which particular differences between pairs of means are significant as post hoc tests. Table 5 shows the results of the MANOVA and Hotelling *T*^2^ test of the classifier corresponding to each evaluation index under EMD and VMD frameworks. As can be seen from Table 5, there is no statistically significant difference between KNN and RF under the VMD framework except *AUC*; there is no statistically significant difference between the classification effect of KNN and RF. And in the EMD framework, DT and LR classifiers show no significant difference in ACC, SEN, NPV, and *AUC*. Additionally, KNN and SVM classifiers exhibit no significant difference in SEN and NPV. These results are consistent with the visual representation in Figure S1.

We applied SHAP values to KNN using the V3 lead under the VMD framework to achieve the best predictive effect and interpretability. The results was shown in Figure S4.

## 4. Discussion

This study aims to explore the electrophysiological signal properties of modes, combining mode decomposition with higher-order spectral analysis to enhance spectral characteristics on the basis of previous studies. The feature extraction framework of the modes is constructed by the application of bispectral analysis. Through performing bispectral analysis on each mode, this study combines high-order spectral features with the nonlinear and frequency domain features of the modes. This systematic approach captures subtle fluctuations, forming a feature vector representing ECG signal characteristics. This vector is then incorporated into a machine learning classifier. In this study, the highest classification accuracy under the EMD framework is 92.54%, while the highest classification accuracy under the VMD framework is 98.30%. This suggests that the proposed feature extraction model in this study is significantly valuable for clinical application.

Under all leads, the discriminative effectiveness of the VMD surpasses that of the EMD. Furthermore, within each mode, the discriminative effectiveness of the VMD outperforms that of the EMD. While the classification performance of the first mode in the EMD is optimal, the classification performance of the second and third modes in the VMD excels. Importantly, the classification performance of the VMD framework consistently outperforms that of the EMD framework within the same mode. Moreover, the lowest average accuracy of the five modes of the VMD framework is always higher than the highest average accuracy of the five modes of the EMD framework.

Among the five classifiers, VMD demonstrates superior discriminative effectiveness compared to EMD. While RF achieves optimal performance under the EMD framework, both RF and KNN outperform it under the VMD framework. Regardless of the classifier, the classification performance under the VMD framework consistently outperforms that under the EMD framework. RF and KNN perform well in ECG analysis mainly due to their advantages with high-dimensional data, nonlinearity, robustness and local pattern recognition, as well as their adaptability to ECG data. ECG are high-dimensional time series data, and RF has a strong processing capacity for high-dimensional data, effectively capturing complex patterns in time series, and demonstrating good robustness to noise and outliers, and can provide robust classification performance for noisy ECG data [41]. Simultaneously, RF can capture nonlinear relationships, and abnormal ECG, such as an arrhythmia, which may involve complex nonlinear relationships, enhancing the classification performance of RF [44,45]. However, the local pattern of ECG signals is crucial for classification, and KNN excels in capturing this local pattern [42]. It can more flexibly adapt to the complex distribution that may exist in ECG signals [46].

In clinical studies, echocardiography showed left ventricular dilation in both DCM and ICM for thoracic leads (V1, V2, V3, V4, V5, V6) and limb leads (I, II, III, aVL, aVF, aVR). Compared with limb leads, thoracic leads have a higher reference value for cardiomyopathy [47]. Under the two decomposition frameworks of this study, the chest lead has a higher accuracy than the limb lead, which verifies the superiority of the chest lead in the diagnosis and treatment of cardiomyopathy.

Among patients with ICM and DCM, the V3 lead possesses the highest differentiating ability compared to the other 11 leads [48]. In this study, the lead V3 reaches the highest accuracy under the VMD framework; conversely, the lead V5 reaches the highest accuracy under the EMD framework. Notably, the V3 lead under the VMD framework exhibits the highest classification accuracy compared to other leads, which is highly consistent with clinical studies. This observation further substantiates the inferiority of the EMD to the VMD in the differential diagnosis of ECG signals.

## 5. Conclusions

This study proposes a feature extraction framework combining ECG signal mode decomposition and bispectral analysis. It establishes a machine learning model capable of accurately and efficiently discriminating between patients with DCM and ICM. Additionally, the study explores the application value of VMD in ECG signals, providing a novel approach to the differential diagnosis of cardiomyopathy using ECG signals. The results show that ECG signals combined with VMD, bispectral analysis, and machine learning can help to distinguish DCM from ICM in clinical practice. Furthermore, this study verified the superior performance of VMD compared with EMD in ECG diagnosis of cardiovascular diseases. These findings contribute valuable reference insights to the field of electrophysiological signal analysis.

## Figures and Tables

**Table 1:** Demographic, clinical, and echocardiographic characteristics of patients with DCM and ICM.

Variable	Dilated Cardiomyopathy (*n* = 44)	Ischemic Cardiomyopathy (*n* = 43)	*p* Value
Age	62.41 ± 12.72	62.49 ± 12.18	0.976
Gender (M), *n* (%)	29 (65.9)	37 (86.0)	0.052
Smoking, *n* (%)	18 (40.9)	28 (65.1)	0.041 *
Alcohol, *n* (%)	8 (18.2)	4 (9.3)	0.374
Hypertension, *n* (%)	16 (36.4)	18 (41.9)	0.760
Diabetes mellitus, *n* (%)	8 (18.2)	18 (41.9)	0.029 *
LVEDD (mm)	63.95 ± 5.65	61.95 ± 6.40	0.126
LVEF (%)	33 ± 7	33 ± 7	0.860
Septal thickness (mm)	10.25 ± 1.30	10.51 ± 1.40	0.369

Note: Data were expressed as means ± standard variance for continuous variables and frequency (percentage%) for count variables. * denotes *p* < 0.05.

**Table 2:** Extracted features and calculation methods.

Feature	Calculation Method
bispectral brightness	$\frac{\sum_{i=F}^{\frac{N}{2}}\sum_{j=F}^{\frac{N}{2}}\left|\omega (i,j)\right|}{\sum_{i=0}^{\frac{N}{2}}\sum_{j=0}^{\frac{N}{2}}\left|\omega (i,j)\right|}$,
bispectral flatness	$\frac{\sqrt[{\frac{N}{2}}]{\sqrt[{\frac{N}{2}}]{\Pi_{i=0}^{\frac{N}{2}}\Pi_{j=0}^{\frac{N}{2}}\left|\omega (i,j)\right|}}}{\frac{1}{N/2}\cdot \frac{1}{N/2}\sum_{i=0}^{\frac{N}{2}}\sum_{j=0}^{\frac{N}{2}}\left|\omega (i,j)\right|}$
bispectral roll-off	$\max F:\sum_{j=0}^{F}\sum_{i=0}^{F}\left|\omega (i,j)\right|\leq \beta \;\cdot \sum_{j=0}^{\frac{N}{2}}\sum_{i=0}^{\frac{N}{2}}\left|\omega (i,j)\right|$, *β* = 0.95
normalized bispectral entropy	$-\sum_{i,j\in N}p_{i,j}*logp_{i,j}$, $p_{i,j}=\frac{\left|\omega (i,j)\right|}{\sum_{i,j\in N}\left|\omega (i,j)\right|}$
normalized bispectral squared entropy	$-\sum_{i,j\in N}q_{i,j}*logq_{i,j}$, $q_{i,j}=\frac{{\left|\omega (i,j)\right|}^{2}}{\sum_{i,j\in N}{\left|\omega (i,j)\right|}^{2}}$
the sum of logarithmic amplitudes of the bispectrum	$\sum_{i,j\in N}\log(\left|\omega \left(i,j\right)\right|)$
the sum of logarithmic amplitudes of diagonal elements in the bispectrum	$\sum_{k\in N}\log(\left|\omega \left(k,k\right)\right|)$
the first-order spectral moment of the amplitudes of diagonal elements in the bispectrum	$\sum_{k\in N}k\log(\left|\omega (k,k)\right|)$
the second-order spectral moment of the amplitudes of diagonal elements in the bispectrum	$\sum_{k\in N}\left(k-\sum_{k\in N}k\log(\left|\omega (k,k)\right|){)}^{2}*\log(\left|\omega (k,k)\right|\right)$
the peak value of Power Spectral Density	the 16-order AR model whose parameters were estimated using Burg’s method
approximate entropy	$\emptyset^{m}\left(r\right)-\emptyset^{m+1}\left(r\right)$,$\;\emptyset^{m}\left(r\right)\;$ is the mean approximation
fuzzy entropy	$-\int \mu \left(x\right)\log\left(\mu \left(x\right)\right)$, $\mu \left(x\right)\;$ is the membership of a fuzzy system
sample entropy	$-\sum \left[P\left(i\right)*\log\left(P\left(i\right)\right)\right]$, $P\left(i\right)=\frac{\sum \left[d\left(x\left(i\right)\right),\;x\left(j\right)\leq r\right],\;i\neq j}{N-m+1}$
permutation entropy	$-\sum \left[P\left(\pi \right)*\log\left(P\left(\pi \right)\right)\right]$
Lempel–Ziv complexity	the new mode rate of the signal is obtained through the iteration of the string

*F* denotes the specified cutoff frequency, set at 120 Hz. $\omega \left(i,j\right)$ denotes the spectral amplitude value at the point (i,j). *N* denotes the number of points of the bispectral matrix.

**Table 3:** Pearson correlation coefficients between the first 10 modes and the original EMD and VMD signals.

Method	Lead	Mode									
		Mode1	Mode2	Mode3	Mode4	Mode5	Mode6	Mode7	Mode8	Mode9	Mode10
EMD	I	0.367	0.522	0.548	0.434	0.270	0.118	0.046	0.018	0.008	0.006
	II	0.373	0.520	0.548	0.407	0.241	0.100	0.033	0.013	0.010	0.006
	III	0.375	0.509	0.559	0.439	0.296	0.121	0.056	0.023	0.012	0.009
	aVR	0.395	0.530	0.547	0.405	0.239	0.079	0.026	0.011	0.006	0.005
	aVL	0.355	0.506	0.549	0.444	0.294	0.120	0.049	0.025	0.012	0.010
	aVF	0.382	0.504	0.557	0.419	0.259	0.103	0.044	0.019	0.012	0.009
	V1	0.387	0.543	0.531	0.360	0.221	0.055	0.025	0.010	0.005	0.005
	V2	0.389	0.480	0.540	0.359	0.215	0.052	0.019	0.007	0.005	0.005
	V3	0.379	0.449	0.524	0.346	0.208	0.048	0.018	0.006	0.004	0.002
	V4	0.355	0.406	0.478	0.363	0.207	0.050	0.021	0.008	0.005	0.006
	V5	0.306	0.413	0.463	0.366	0.213	0.051	0.016	0.008	0.006	0.007
	V6	0.349	0.455	0.486	0.375	0.205	0.067	0.021	0.008	0.005	0.005
VMD	I	0.580	0.690	0.506	0.373	0.257	0.167	0.103	0.066	0.043	0.029
	II	0.589	0.708	0.482	0.353	0.246	0.162	0.102	0.065	0.042	0.026
	III	0.632	0.694	0.466	0.334	0.234	0.149	0.096	0.063	0.041	0.029
	aVR	0.557	0.707	0.501	0.372	0.261	0.172	0.109	0.067	0.042	0.026
	aVL	0.608	0.692	0.485	0.350	0.247	0.164	0.103	0.068	0.044	0.031
	aVF	0.599	0.705	0.463	0.347	0.246	0.159	0.103	0.066	0.044	0.028
	V1	0.524	0.693	0.545	0.406	0.281	0.178	0.112	0.068	0.044	0.028
	V2	0.533	0.721	0.545	0.390	0.249	0.158	0.094	0.056	0.035	0.022
	V3	0.585	0.707	0.511	0.375	0.243	0.151	0.094	0.060	0.037	0.022
	V4	0.643	0.703	0.461	0.337	0.225	0.146	0.094	0.061	0.039	0.024
	V5	0.603	0.687	0.499	0.368	0.261	0.171	0.107	0.070	0.046	0.027
	V6	0.568	0.681	0.523	0.396	0.277	0.184	0.115	0.074	0.047	0.032

**Table 4:** The leads used by the five classifiers to achieve the highest accuracy under VMD and EMD.

Classifier	EMD		VMD	
	Lead	Accuracy	Lead	Accuracy
DT	V4	73.74 ± 2.03	V3	88.89 ± 1.57
KNN	V4	83.62 ± 1.64	V3	98.30 ± 0.58
LR	I	73.03 ± 2.24	V3	81.53 ± 1.08
RF	V5	92.54 ± 1.41	V5	98.23 ± 0.55
SVM	V4	81.91 ± 1.28	V3	95.58 ± 0.54

**Table 5:** The results of the Hotelling *T^2^* test of the classifier corresponding to each evaluation index.

Decomposition	Index	*F*	*p*	Classifiers (Best → Worst)
EMD	*ACC*	634.667	<0.01	RF; KNN; SVM; DT, LR;
	*SEN*	90.425	<0.01	RF; KNN; SVM; LR, DT;
	*SPE*	142.984	<0.01	RF; KNN; SVM; DT; LR;
	*PPV*	201.938	<0.01	RF; KNN; SVM; DT; LR;
	*NPV*	190.536	<0.01	RF; KNN, SVM; LR, DT;
	*AUC*	307.866	<0.01	RF; KNN; SVM; LR, DT;
VMD	*ACC*	232.490	<0.01	RF, KNN; SVM; DT; LR;
	*SEN*	159.015	<0.01	KNN, RF; SVM; DT; LR;
	*SPE*	80.986	<0.01	RF, KNN; SVM; DT; LR;
	*PPV*	155.806	<0.01	RF, KNN; SVM; DT; LR;
	*NPV*	186.085	<0.01	KNN, RF; SVM; DT; LR;
	*AUC*	106.898	<0.01	RF; KNN; SVM; DT; LR;

Note: The classifiers are arranged according to the classification effect from the best to the worst. The two classifiers where the null hypothesis of equal population mean vectors is not rejected with *p* > 0.05 after Bonferroni correction are underlined.

## Data Availability

The data presented in this study are available on request from the corresponding author. The data are not publicly available due to issues of participant confidentiality.

## Supplementary Materials

The following supporting information can be downloaded at: https://www.mdpi.com/article/10.3390/bioengineering11101012/s1, Figure S1: Classification effect radar chart of each lead; Figure S2: The grouping box diagram of the average accuracy of each mode of 12 leads under EMD; Figure S3: The grouping box diagram of the average accuracy of each mode of 12 leads under VMD; Figure S4: The model’s interpretation; Table S1: Eigenvalues and contribution rates under EMD and VMD frameworks; Table S2: Classification performance of 5 classifiers under VMD framework; Table S3: Classification performance of 5 classifiers under EMD framework.
