# Supplementary material for: Comparison of Electrocardiogram between Dilated Cardiomyopathy and Ischemic Cardiomyopathy Based on Empirical Mode Decomposition and Variational Mode Decomposition
Source: Bioengineering (Basel). 2024 Oct 11;11(10):1012. doi: 10.3390/bioengineering11101012 (PMC11505311; doi:10.3390/bioengineering11101012)
Supplement: Supplementary file 1 [file bioengineering-11-01012-s001.zip › bioengineering-3232653-supplementary.pdf]

Table S1 Eigenvalues and contribution rates under EMD and VMD frameworks

Order	EMD		VMD	
	eigenvalues	Accumulated contributions (%)	eigenvalues	Accumulated contributions (%)
1	0.0086	85.33	0.0019	71.44
2	0.0007	92.12	0.0006	94.94
3	0.0004	95.85	0.0001	98.00
4	0.0002	98.08	0.0000	99.28
5	0.0001	99.10	0.0000	99.61
6	0.0000	99.56	0.0000	99.82
7	0.0000	99.88	0.0000	99.94
8	0.0000	99.98	0.0000	99.98
9	0.0000	100.00	0.0000	100.00
10	0.0000	100.00	0.0000	100.00

Table S2 Classification performance of 5 classifiers under VMD framework

classfier	lead	*ACC(%)*	*SEN(%)*	*SPE(%)*	*PPV(%)*	*NPV(%)*	*AUC*
	I	82.53±0.92	81.17±3.22	83.68±2.29	79.64±2.61	84.98±2.61	0.87±0.01
	II	77.37±1.60	80.19±6.20	75.18±4.30	72.13±3.08	82.91±3.99	0.82±0.02
	III	77.32±1.64	78.53±4.26	76.35±4.94	72.90±4.33	81.76±2.14	0.82±0.02
	aVR	83.55±1.84	81.15±4.18	85.42±3.90	81.70±3.07	85.26±2.89	0.89±0.01
	aVL	79.40±1.92	83.04±4.00	76.45±3.42	73.79±2.61	85.14±2.50	0.85±0.02
	aVF	78.40±2.41	74.10±3.05	81.73±4.54	76.62±3.58	79.91±2.17	0.83±0.02
DT	V1	84.66±1.67	81.43±3.54	87.26±3.79	83.66±3.80	85.62±2.35	0.88±0.02
	V2	84.97±1.55	83.82±3.16	85.85±3.23	82.44±3.38	87.15±2.06	0.89±0.02
	V3	**88.89±1.57**	87.35±3.42	90.23±2.17	87.55±2.66	90.03±3.04	0.91±0.01
	V4	85.72±1.61	83.72±2.55	87.35±2.48	84.08±2.74	87.08±2.14	0.89±0.02
	V5	88.07±1.33	84.37±1.31	91.03±1.98	88.20±2.77	87.95±1.42	0.90±0.01
	V6	83.97±1.35	84.36±3.97	83.63±3.55	80.84±2.81	86.97±2.50	0.88±0.02
	I	96.57±0.59	96.40±1.77	96.67±1.41	95.88±1.52	97.19±1.30	0.99±0.00
	II	96.47±0.47	96.68±1.48	96.30±1.57	95.45±1.84	97.36±1.13	0.99±0.00
	III	95.98±0.65	96.51±1.88	95.61±1.74	94.64±2.07	97.14±1.56	0.99±0.00
	aVR	96.54±0.61	96.44±1.73	96.66±0.91	95.79±1.16	97.15±1.42	0.99±0.00
	aVL	96.21±0.54	95.61±1.42	96.67±1.26	95.82±1.56	96.55±1.04	0.99±0.00
	aVF	95.98±0.51	96.45±1.49	95.59±1.64	94.64±1.79	97.17±1.12	0.99±0.00
KNN	V1	96.61±0.43	96.45±1.42	96.74±1.11	95.94±1.28	97.17±1.15	0.99±0.00
	V2	97.87±0.76	97.64±0.97	98.09±1.35	97.56±1.75	98.12±0.81	0.99±0.00
	V3	**98.30±0.58**	98.35±0.92	98.27±0.75	97.80±0.97	98.68±0.81	0.99±0.00
	V4	98.18±0.32	97.94±1.04	98.37±0.81	97.95±1.01	98.37±0.83	1.00±0.00
	V5	97.90±0.60	98.00±0.90	97.79±1.11	97.27±1.30	98.43±0.64	0.99±0.00
	V6	96.46±0.58	95.66±1.99	97.08±1.33	96.41±1.57	96.57±1.41	0.99±0.00
	I	78.55±1.71	82.21±3.42	75.65±4.35	72.81±2.87	84.46±2.68	0.86±0.02
	II	73.50±1.83	71.87±7.35	74.79±6.00	69.73±3.22	77.27±3.95	0.80±0.02
	III	74.94±1.89	76.81±4.00	73.59±6.06	70.24±5.32	79.94±2.11	0.81±0.02
	aVR	80.72±1.20	80.31±2.09	81.02±1.96	77.01±1.38	83.90±1.72	0.87±0.01
	aVL	76.91±1.19	79.59±3.16	74.71±2.79	71.49±2.36	82.27±1.41	0.83±0.01
	aVF	75.32±1.52	81.72±4.47	70.25±5.24	68.73±3.41	83.11±2.60	0.82±0.01
LR	V1	78.45±1.86	74.74±4.62	81.24±4.61	76.25±3.29	80.35±2.02	0.82±0.01
	V2	79.48±1.48	83.63±1.52	76.17±3.04	73.43±2.60	85.56±1.26	0.86±0.02
	V3	**81.53±1.08**	77.15±3.09	85.02±3.44	80.31±3.59	82.58±2.13	0.87±0.01
	V4	79.55±1.59	78.71±2.97	80.14±4.46	76.17±3.09	82.61±1.66	0.86±0.01
	V5	78.70±1.40	79.87±4.21	77.78±3.19	74.18±2.48	82.99±3.09	0.83±0.01
	V6	80.79±0.95	78.49±4.15	82.55±3.12	78.64±1.78	82.72±1.97	0.86±0.01
	I	96.54±0.73	96.69±0.85	96.43±1.14	95.54±1.27	97.34±0.85	0.99±0.00
	II	96.06±0.61	95.61±1.72	96.42±1.28	95.51±1.53	96.51±1.33	0.99±0.00
RF	III	96.24±0.67	96.27±1.48	96.18±1.28	95.33±1.40	97.03±1.05	0.99±0.00
	aVR	96.91±0.63	96.12±1.25	97.53±1.12	96.89±1.29	96.96±0.98	0.99±0.00
	aVL	96.12±0.94	95.87±1.95	96.32±1.60	95.43±1.96	96.74±1.41	0.99±0.00

	aVF	95.61±0.63	96.27±1.26	95.10±1.49	93.98±1.73	97.00±0.96	0.99±0.00
	V1	97.36±0.69	97.54±1.13	97.19±1.83	96.56±2.03	98.06±0.86	1.00±0.00
	V2	97.81±0.60	97.62±1.01	97.96±0.88	97.40±1.20	98.13±0.76	1.00±0.00
	V3	98.06±0.41	98.12±1.19	98.05±0.71	97.51±0.98	98.48±1.02	1.00±0.00
	V4	98.05±0.55	97.71±0.94	98.33±0.92	97.92±1.05	98.16±0.82	1.00±0.00
	V5	**98.23±0.55**	98.02±0.88	98.39±0.85	98.01±1.01	98.42±0.71	1.00±0.00
	V6	96.76±0.92	96.20±1.99	97.21±1.50	96.54±1.91	96.99±1.51	0.99±0.00
SVM	I	93.06±0.93	92.95±1.12	93.10±2.14	91.47±2.00	94.40±0.83	0.97±0.01
	II	92.65±1.36	92.82±2.05	92.52±2.71	90.95±2.78	94.17±1.71	0.97±0.01
	III	91.77±1.05	90.78±2.59	92.60±2.74	90.87±3.06	92.64±1.91	0.97±0.01
	aVR	93.47±0.88	93.41±1.78	93.52±1.58	91.98±1.62	94.73±1.43	0.98±0.00
	aVL	91.62±0.95	92.47±1.61	90.92±2.12	89.07±2.10	93.86±1.10	0.97±0.01
	aVF	92.25±1.05	92.48±2.06	92.12±1.53	90.30±1.96	93.89±1.78	0.97±0.01
	V1	93.31±1.08	92.03±2.89	94.37±1.58	92.83±2.03	93.74±2.28	0.98±0.01
	V2	95.70±0.88	94.34±2.10	96.78±1.18	95.83±1.58	95.62±1.59	0.99±0.00
	V3	**95.85±0.54**	95.78±1.83	95.96±1.26	94.88±1.58	96.62±1.64	0.99±0.00
	V4	94.87±1.17	94.99±2.16	94.77±2.44	93.63±2.71	95.99±1.68	0.99±0.00
	V5	94.14±1.33	93.60±2.74	94.50±2.81	93.38±2.73	94.93±1.95	0.98±0.01
	V6	92.99±0.99	92.28±1.68	93.56±2.07	92.12±2.40	93.77±1.12	0.98±0.01

Note: The leads with the highest accuracy under each classifier are highlighted in bold.

Table S3 Classification performance of 5 classifiers under EMD framework

classifier	lead	*ACC(%)*	*SEN(%)*	*SPE(%)*	*PPV(%)*	*NPV(%)*	*AUC*
DT	I	73.60±3.21	71.00±8.94	75.18±10.58	71.02±6.74	76.94±2.79	0.79±0.02
	II	68.18±1.65	69.60±7.99	66.96±7.75	62.96±3.40	73.91±2.79	0.72±0.02
	III	69.47±2.20	69.48±9.64	69.32±7.43	64.77±3.54	74.57±4.04	0.73±0.02
	aVR	70.54±1.73	64.42±8.35	75.52±5.79	68.04±4.24	72.99±3.80	0.75±0.02
	aVL	71.18±2.38	67.63±5.77	74.03±4.91	67.92±4.22	74.07±2.66	0.76±0.03
	aVF	66.27±2.20	57.03±11.78	73.59±10.16	64.02±4.29	68.85±4.09	0.68±0.03
	V1	71.64±1.36	66.27±4.57	76.03±4.60	69.01±4.01	73.89±2.64	0.76±0.02
	V2	68.57±2.14	61.76±7.16	73.93±7.49	65.88±4.87	71.09±3.14	0.72±0.02
	V3	69.04±2.48	73.56±10.38	65.25±9.68	63.39±4.68	76.38±4.66	0.74±0.02
	V4	**73.74±2.08**	65.54±5.45	80.28±6.18	72.86±5.52	74.79±2.54	0.76±0.02
	V5	72.67±1.53	71.84±6.88	73.56±4.76	68.58±3.08	76.80±4.57	0.76±0.02
	V6	71.75±1.97	73.76±9.84	69.95±9.92	67.00±5.94	77.88±4.06	0.78±0.02
KNN	I	81.28±1.27	80.14±3.42	82.13±3.46	78.28±2.97	83.92±2.24	0.89±0.01
	II	79.13±2.13	80.70±4.89	77.75±6.97	74.78±4.38	83.78±2.27	0.87±0.02
	III	81.95±1.56	83.78±4.24	80.44±4.79	77.65±3.68	86.31±2.34	0.89±0.01
	aVR	81.87±0.83	80.92±2.11	82.65±2.87	78.89±2.59	84.46±1.52	0.89±0.01
	aVL	81.34±1.48	83.44±5.56	79.72±5.63	77.21±4.35	85.88±3.61	0.89±0.01
	aVF	79.83±1.58	77.45±4.00	81.72±3.42	77.22±2.70	82.10±2.92	0.87±0.02
	V1	79.21±1.54	75.90±8.07	81.97±6.56	77.60±5.07	81.38±4.76	0.87±0.01
	V2	81.40±1.88	83.16±4.27	80.01±4.66	76.95±3.78	85.80±2.98	0.89±0.01
	V3	81.72±1.33	79.29±3.26	83.63±3.57	79.58±3.39	83.57±1.97	0.89±0.01
	V4	**83.62±1.64**	87.00±2.08	80.90±3.40	78.33±2.89	88.80±1.34	0.91±0.01
	V5	80.79±1.37	78.23±3.90	82.81±4.33	78.65±3.67	82.77±2.16	0.88±0.01
	V6	81.04±1.37	81.32±5.37	80.82±4.01	77.19±3.41	84.76±3.18	0.88±0.01
LR	I	**73.03±2.24**	75.05±6.37	71.63±7.86	68.24±5.50	78.54±3.80	0.79±0.01
	II	70.79±1.48	72.60±6.79	69.40±5.08	65.48±2.60	76.37±3.49	0.75±0.02
	III	67.95±1.91	73.93±5.44	63.22±5.54	61.78±3.05	75.39±2.83	0.74±0.02
	aVR	70.41±1.30	73.37±6.66	67.92±6.52	64.90±2.69	76.51±2.83	0.76±0.01
	aVL	70.66±2.38	74.18±7.11	68.07±8.12	65.66±5.30	76.84±3.78	0.77±0.02
	aVF	65.87±2.45	70.92±7.19	61.94±7.39	59.88±3.59	73.14±3.84	0.71±0.02
	V1	69.93±1.78	72.86±5.35	67.80±5.48	64.53±3.68	75.90±3.92	0.75±0.02
	V2	68.92±2.51	63.99±6.74	72.91±6.33	65.38±4.85	72.07±3.54	0.73±0.03
	V3	66.31±1.66	72.73±6.48	61.32±6.53	60.16±3.37	74.04±4.14	0.72±0.02
	V4	72.56±2.25	69.79±8.04	74.86±8.56	69.39±5.79	76.13±3.35	0.79±0.02
	V5	69.63±1.70	68.56±7.80	70.60±7.28	65.45±4.04	74.09±3.71	0.75±0.02
	V6	70.32±1.58	74.85±6.66	67.03±6.85	64.40±4.12	77.43±4.14	0.76±0.01
RF	I	90.39±1.37	88.23±3.21	92.02±2.52	89.98±2.35	90.84±2.12	0.96±0.01
	II	89.04±1.17	88.99±3.61	89.13±3.26	86.78±3.37	91.15±2.59	0.96±0.00
	III	89.66±1.92	89.10±3.08	90.14±4.43	88.08±4.69	91.26±2.07	0.96±0.01
	aVR	90.28±0.92	92.57±2.15	88.44±1.68	86.50±1.61	93.76±1.62	0.96±0.01
	aVL	91.29±1.17	91.72±2.53	90.93±1.70	89.09±1.93	93.22±1.75	0.97±0.01
	aVF	88.03±1.65	88.75±3.35	87.54±2.01	84.95±2.42	90.72±2.84	0.95±0.01

	V1	90.28±0.63	90.10±2.70	90.44±2.33	88.34±2.49	92.00±2.09	0.96±0.01
	V2	90.03±1.15	89.55±2.67	90.33±3.06	88.21±2.59	91.69±1.80	0.96±0.01
	V3	90.98±1.05	92.23±3.85	90.03±3.11	88.18±3.43	93.65±2.81	0.97±0.00
	V4	90.63±1.10	91.06±2.28	90.23±1.19	88.02±1.50	92.85±1.26	0.97±0.01
	V5	**92.54±1.41**	91.69±1.96	93.27±2.58	91.62±3.09	93.33±1.73	0.98±0.01
	V6	92.50±0.84	90.47±3.14	94.17±1.92	92.48±2.33	92.60±2.48	0.97±0.01
SVM	I	80.94±1.05	82.49±4.71	79.76±3.72	76.53±3.43	85.26±3.30	0.87±0.01
	II	76.47±2.03	80.52±6.24	73.29±5.00	70.70±3.59	82.89±4.20	0.84±0.02
	III	78.49±1.08	81.65±4.89	75.98±4.96	73.35±3.50	84.02±2.86	0.86±0.01
	aVR	80.93±1.30	81.74±3.02	80.29±3.32	76.89±3.09	84.70±2.08	0.88±0.01
	aVL	78.78±2.03	79.08±2.44	78.58±4.58	75.06±4.01	82.31±1.80	0.85±0.02
	aVF	76.25±1.70	79.05±3.58	74.02±3.49	70.77±2.73	81.74±2.50	0.83±0.02
	V1	76.25±1.65	77.95±6.61	74.71±6.60	71.54±3.43	81.33±3.18	0.83±0.01
	V2	75.94±1.91	78.93±4.69	73.54±4.31	70.32±3.53	81.70±3.06	0.84±0.02
	V3	75.47±1.17	74.36±5.80	76.38±6.51	71.94±4.72	79.11±3.07	0.82±0.01
	V4	**81.91±1.28**	81.49±3.53	82.18±2.93	78.40±2.38	85.00±2.31	0.89±0.01
	V5	78.74±0.97	78.06±5.15	79.23±3.62	75.14±2.16	82.11±3.00	0.86±0.01
	V6	77.72±1.50	80.21±6.05	75.99±6.57	72.89±5.30	83.14±4.00	0.85±0.01

Note: The leads with the highest accuracy under each classifier are highlighted in bold.

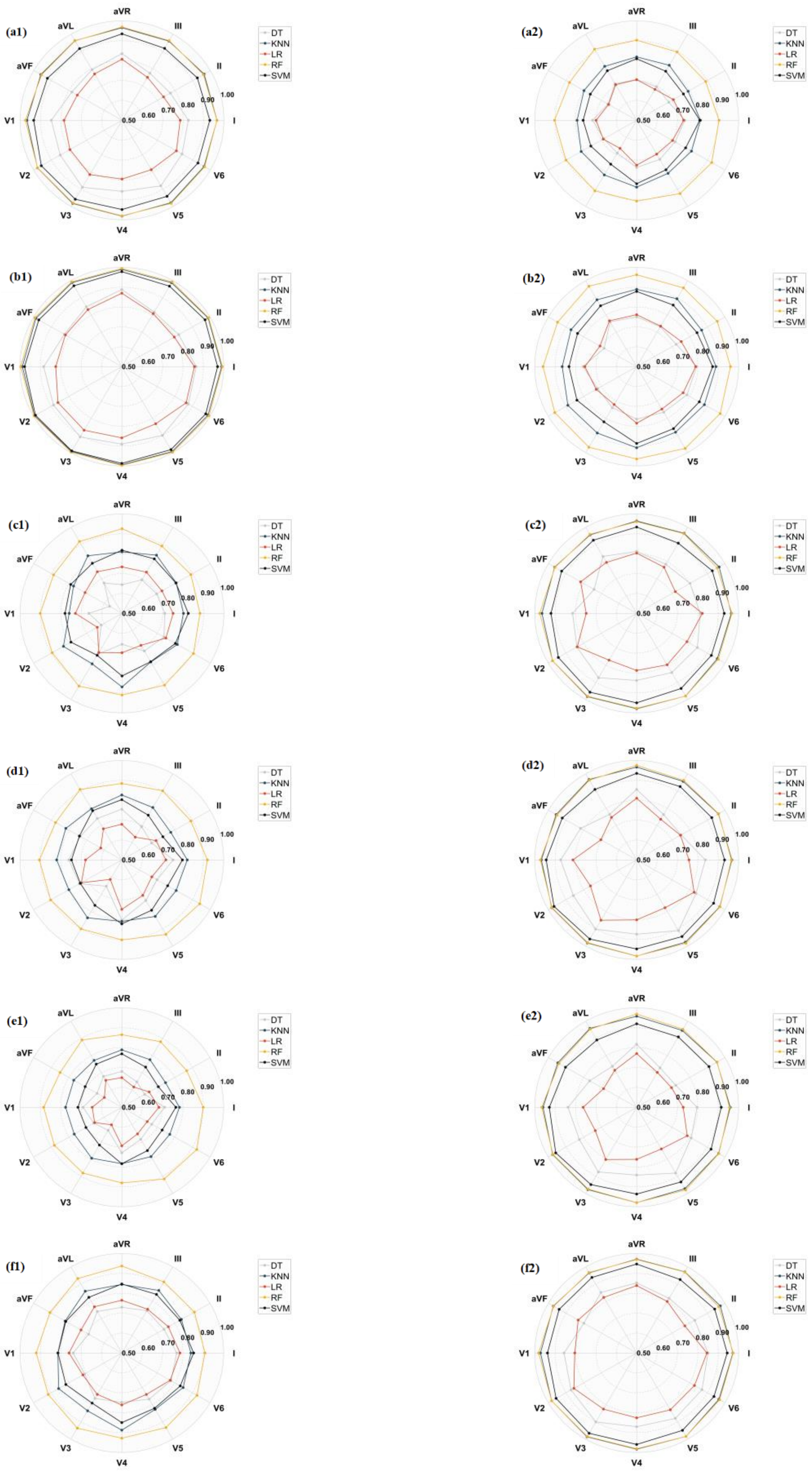
(a1)
(a2)
(b1)
(b2)
(c1)
(c2)
(d1)
(d2)
(e1)
(e2)
(f1)
(f2)
aVR
III
II
I
V6
V5
V4
V3
V2
V1
aVF
aVL
0.50
0.60
0.70
0.80
0.90
1.00
DT
KNN
LR
RF
SVM

Figure S1 Classification effect radar chart of each lead.
(a1) to (f1) are ACC, SEN, SPE, PPV, NPV, AUC of EMD;
(a2) to (f2) are ACC, SEN, SPE, PPV, NPV, AUC of VMD.

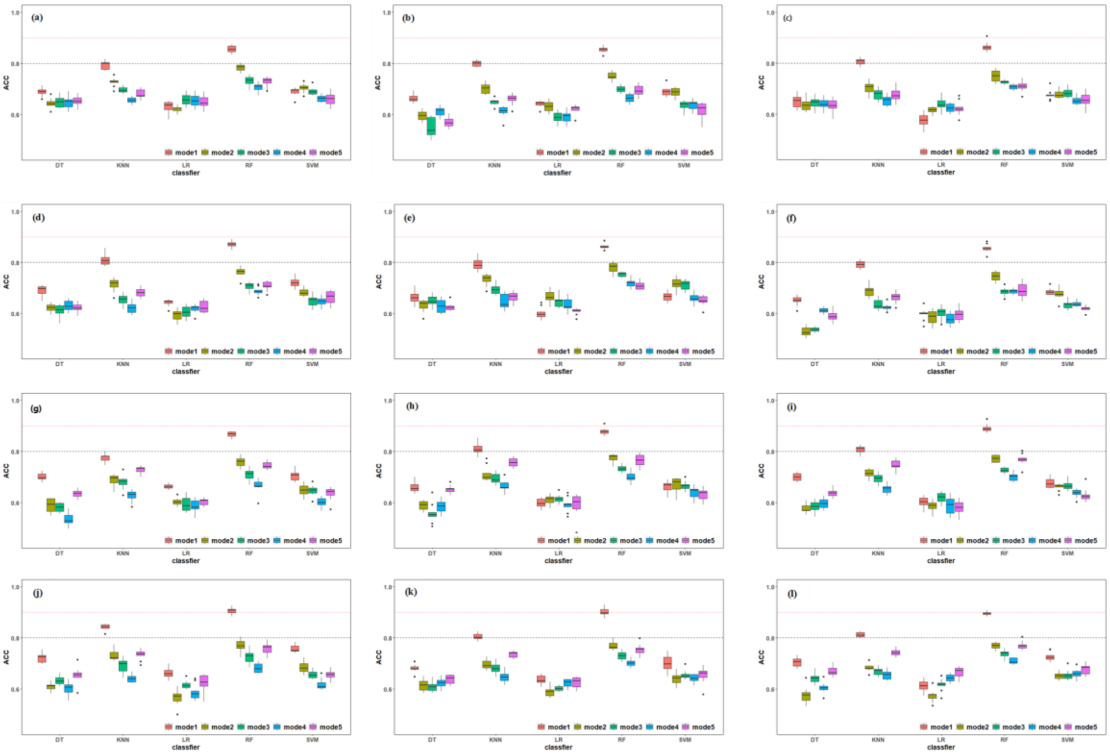

Figure S2 The grouping box diagram of the average accuracy of each mode of 12 leads under EMD. (a) to (l) are lead I, II, III, aVL, aVF, aVR, V1, V2, V3, V4, V5, V6.

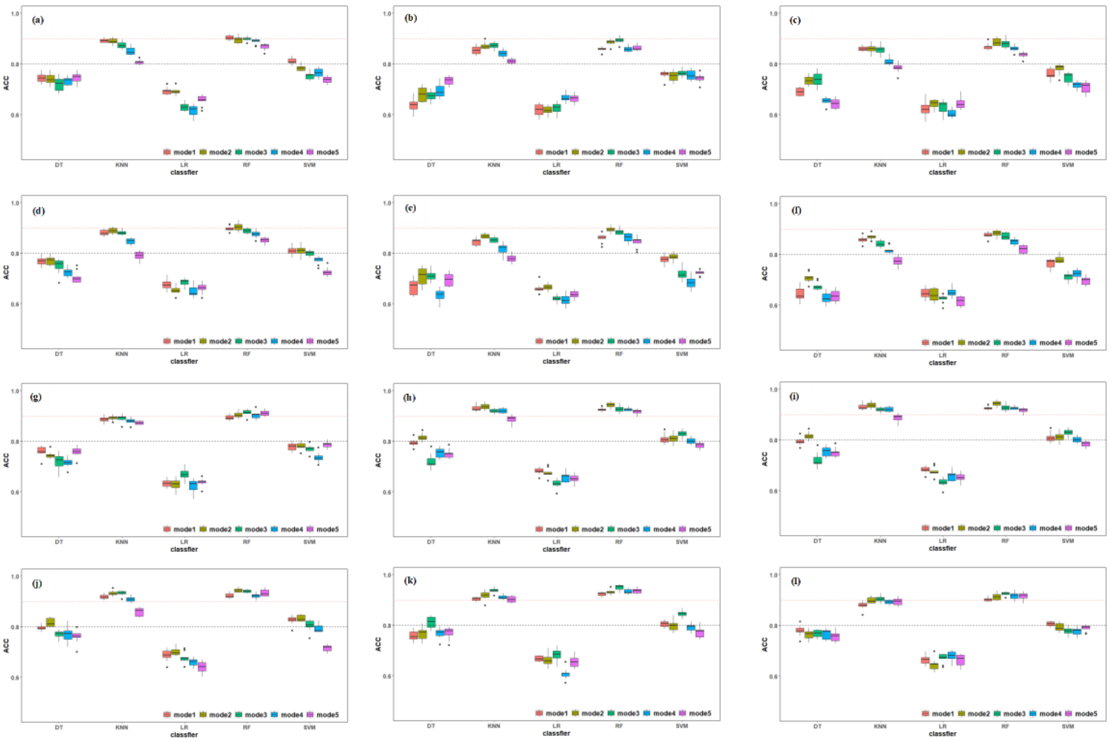

Figure S3 The grouping box diagram of the average accuracy of each mode of 12 leads under VMD. (a) to (l) are lead I, II, III, aVL, aVF, aVR, V1, V2, V3, V4, V5, V6.

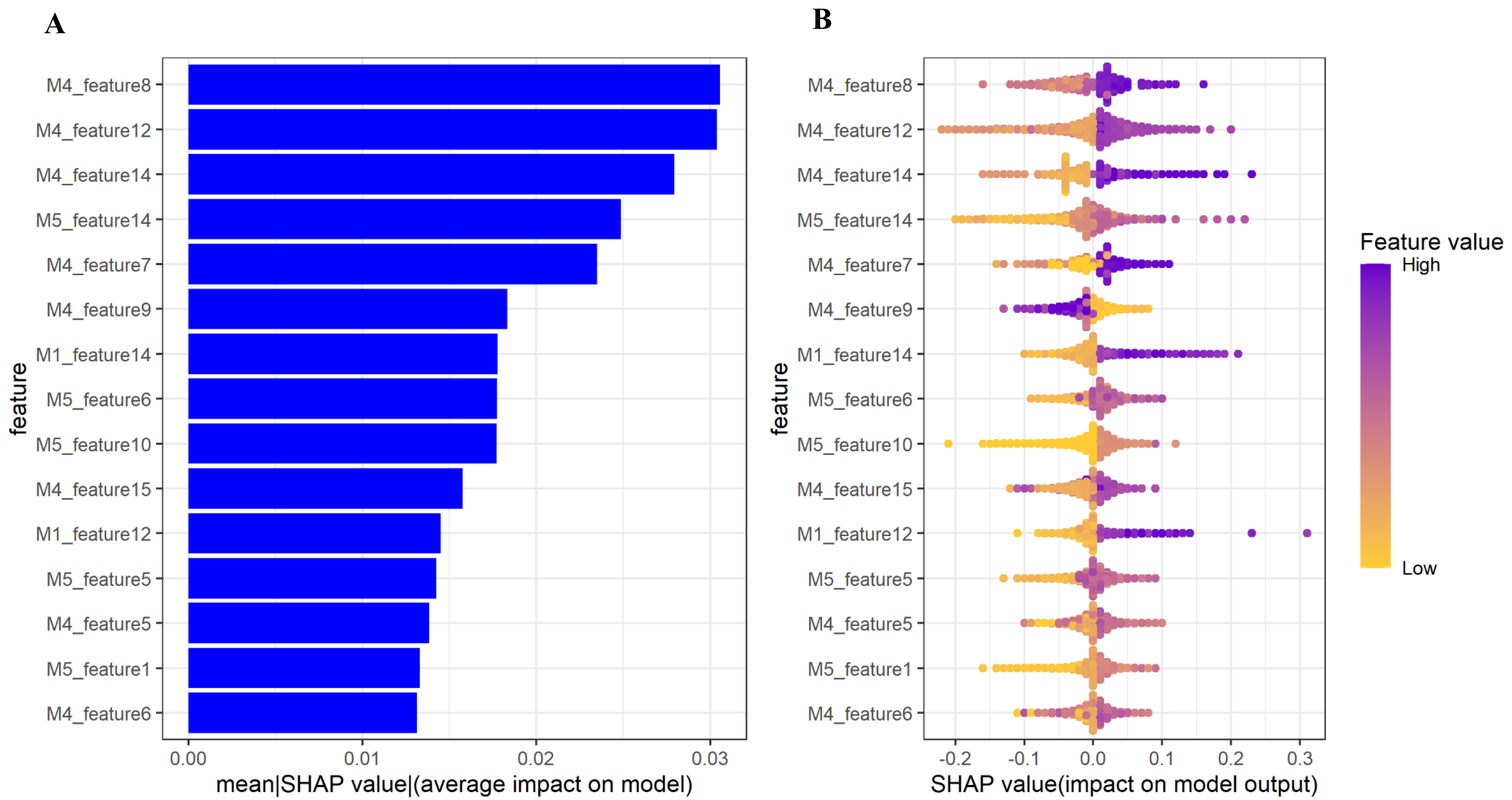

Figure S4. The model's interpretation. (A): The importance ranking of the top 15 features according to the mean (|SHAP value|); (B): The importance ranking of the top 15 features using the optimal model. The higher SHAP value of a feature is given, the higher probability of ICM the patient would have. The purple part in feature value represents higher value.

Note: M denotes mode, with M1 representing mode1, and M4_feature6 referring to feature6 extracted from mode4, and so on.
feature1, the sum of logarithmic amplitudes of the bispectrum; feature5, normalized bispectral entropy; feature6, normalized bispectral squared entropy; feature7, bispectral roll-off; feautre8, bispectral brightness; feature9, bispectral flatness; feature10, power spectral density; feature12, sample entropy; feature14, fuzzy entropy.
